# Urgent Need to Focus on Gender‐Diverse Adolescents in Mental Health Prevention Research: A Cross‐Sectional Comparative Study

**DOI:** 10.1002/hsr2.71500

**Published:** 2025-11-12

**Authors:** Sabrina Mittermeier, Sarah Franke, Linda M. Bonnekoh, Georg Romer, Marcel Romanos, Nikolaus Kleindienst, Arne Buerger

**Affiliations:** ^1^ Department of Child and Adolescent Psychiatry, Psychosomatics and Psychotherapy, Center of Mental Health University Hospital of Wuerzburg Wuerzburg Germany; ^2^ German Center of Prevention Research in Mental Health University of Wuerzburg Wuerzburg Germany; ^3^ Department of Child and Adolescent Psychiatry, Psychosomatics and Psychotherapy University of Muenster Muenster Germany; ^4^ Institute of Psychosomatic Medicine and Psychotherapy, Medical Faculty Mannheim, Central Institute of Mental Health Mannheim Heidelberg University Heidelberg Germany

**Keywords:** adolescence, emotional dysregulation, gender diverse, prevention, suicidality

## Abstract

**Background and Aims:**

Gender‐diverse adolescents—those whose gender identity differs from their assigned sex at birth or who do not conform to traditional binary gender norms—are at significantly higher risk for mental health issues, including suicidality and non‐suicidal self‐injury (NSSI). However, most prevention efforts remain focused on cisgender youth. This study aims to directly compare levels of emotional dysregulation, depressive symptoms, suicidality, and NSSI between gender‐diverse adolescents and their cisgender peers to inform gender‐sensitive prevention strategies.

**Methods:**

This cross‐sectional analysis was conducted in a school‐based sample with participants aged 8–15 years. Gender identity was self‐reported through self‐categorization using a single item with three response options (female, male, diverse). Emotional dysregulation (DERS‐SF), depressive symptoms (PHQ‐9), suicidality (PSS), and NSSI (DSHI‐9) were assessed. Group comparisons between gender‐diverse adolescents (*n* = 13), female‐identifying adolescents (*n* = 1102), and male‐identifying adolescents (*n* = 1045) were performed using Mann‐Whitney U, Fisher's exact, and χ² tests with Bonferroni‐adjusted p‐values.

**Results:**

Compared to female‐identifying and male‐identifying adolescents, gender‐diverse adolescents showed significantly higher emotional dysregulation (vs. females: *p* < 0.001; vs. males: *p* < 0.001) and depressive symptom severity (vs. females: *p* < 0.001; vs. males: *p* < 0.001). Recent suicidality (past two weeks) was markedly elevated in gender‐diverse youth (vs. females: *p* < 0.001; vs. males: *p* < 0.001), although lifetime suicidality did not differ significantly. NSSI behaviors were consistently more severe in the gender‐diverse group, with significantly higher lifetime prevalence, greater frequency, and a broader range of methods (all comparisons *p* < 0.001 vs. both comparison groups).

**Conclusion:**

Gender‐diverse adolescents exhibit substantially higher levels of emotional and behavioral distress compared to both female‐identifying and male‐identifying adolescents. These findings highlight the urgent need for inclusive mental health screening and targeted prevention programs that address the distinct psychological profiles and elevated risks within gender‐diverse youth populations.

## 1 Introduction

Gender diversity encompasses a spectrum of individual experiences of one's gender that may not necessarily partially or fully align with sex assigned at birth, representing an umbrella term for various subjective gender experiences that extend beyond conformity to traditional societal gender norms [1]. Gender adolescents show alarmingly high levels of suicidality [2, 3, 4]. But most suicide prevention measures are targeted at female‐identifying and male‐identifying adolescents. In a previous study on emotional dysregulation (EmD), non‐suicidal self‐injury (NSSI), and suicidality in a representative sample of *n* = 2350 high school students (aged 11–14 years), we emphasized the importance of gender‐dependent foci for future prevention measures [5]. However, due to the limited number of gender‐diverse adolescents in our sample (*n* = 13), we had to exclude them from our suicide risk model, as we could not meaningfully include them in our statistical model without potentially causing bias through forced binary gender categorization.

It is important to gather data from adolescents who identify as gender‐diverse, as these adolescents are known to be a high‐risk group not only for suicidality but also for mental health problems and poorer quality of life in general [6, 7, 8]. Moreover, adolescence is a highly sensitive phase in the development of gender identity [2]. Such data should shed light on the urgent need for targeted prevention measures for gender‐diverse adolescents and foster awareness both among clinicians and the public. This short communication aims to expand the current evidence on psychopathological distress—particularly regarding depression, suicidality, and non‐suicidal self‐injury—in gender‐diverse adolescents. In doing so, we seek to promote gender‐sensitive prevention research and to support the development and implementation of preventive interventions that meet the specific needs of gender‐diverse youth.

## 2 Methods

The present cross‐sectional data were collected as part of a school‐based universal prevention study on emotion regulation in adolescents, called DUDE (“DUDE—Du und deine Emotionen/You and your emotions”); for details, see [5]. The adolescents were aged between 8 and 15 years (gender diverse: *M* = 12.34, SD = 1.19; female‐identifying: *M* = 12.31, SD = 0.69; male‐identifying: *M* = 12.43, SD = 0.72; see Table S1 in the supporting information). Informed consent was obtained from all participants and from their legal guardians. Participants' gender identity was recorded based on self‐disclosure (response options: male, female, gender diverse). EmD was assessed using the 18‐item *Difficulties in Emotion Regulation Scale–Short Form* (DERS‐SF), with higher sum scores reflecting higher EmD. To assess depressive symptoms, we employed the nine‐item *Patient Health Questionnaire* (PHQ‐9); items refer to the past 2 weeks, and higher scores reflect greater depressive symptom severity. We further assessed suicidality using the five‐item *Paykel Suicide Scale* (PSS), which measures the severity of suicidality, encompassing thoughts of death, suicidal ideation, and suicide attempts, referring to the past 2 weeks and lifetime; higher scores reflect more severe suicidality. Finally, we assessed possible NSSI using the nine‐item *Deliberate Self Harm Inventory* (DSHI‐9), which measures lifetime prevalence, frequency, and methods of NSSI. All questionnaires employed in this study have been validated and are frequently used in mainly female‐/male‐identifying adolescents worldwide [5].

Sum scores were calculated for all questionnaires, and incomplete questionnaires were excluded from the analyzes. Due to different group sizes and a lack of normal distribution of the data, we performed two‐sided *Mann‐Whitney U tests, Fisher's exact tests*, and *χ² tests* for group comparisons and report *Bonferroni‐adjusted p‐values* with the a priori significance level of α < 0.05. We performed direct pairwise Mann‐Whitney U tests rather than a Kruskal‐Wallis omnibus test due to the extreme group size imbalance (gender‐diverse *n* = 13 vs. comparison groups *n* > 1000), which would severely reduce test power for detecting differences involving the small group and compromise the chi‐square approximation reliability. This analytical approach extends our previously published framework [5], where we established separate models for emotional dysregulation and suicide risk in female‐identifying and male‐identifying adolescents. The pairwise comparisons allow us to specifically examine how gender‐diverse adolescents compare to each comparison group, informing whether they align with existing models or require separate theoretical consideration. Bonferroni correction was applied to control for multiple testing. All descriptive analyzes were conducted using R Studio (version R 4.1.2).

The DUDE study was ethically approved by both the ethics committee in Wuerzburg (127/19‐me) and the Ministry of Education and Cultural Affairs (IV.7‐BO5106/200/12).

## 3 Results

Group comparisons between gender‐diverse (d) and female‐identifying (f) and male‐identifying (m) adolescents showed significant differences in EmD (d vs. f: *U* = 2488.0, *p* < 0.001; d vs. m: *U* = 1556.6, *p* < 0.001), depressive symptoms (d vs. f: *U* = 3064.0, *p* < 0.001; d vs. m: *U* = 2267.5, *p* < 0.001), suicidality in the past 2 weeks (d vs. f: *U* = 3931.0, *p* < 0.001; d vs. m: *U* = 3333.0, *p* < 0.001), NSSI frequency (d vs. f: *U* = 3748.0, *p* < 0.001; d vs. m: *U* = 3366.5, *p* < 0.001), NSSI methods (d vs. f: *U* = 3823.0, *p* < 0.001; d vs. m: *U* = 3424.0, *p* < 0.001), and NSSI lifetime prevalence (d vs. f: *p* < 0.001; d vs. m: *p* < 0.001).

**Figure 1:**
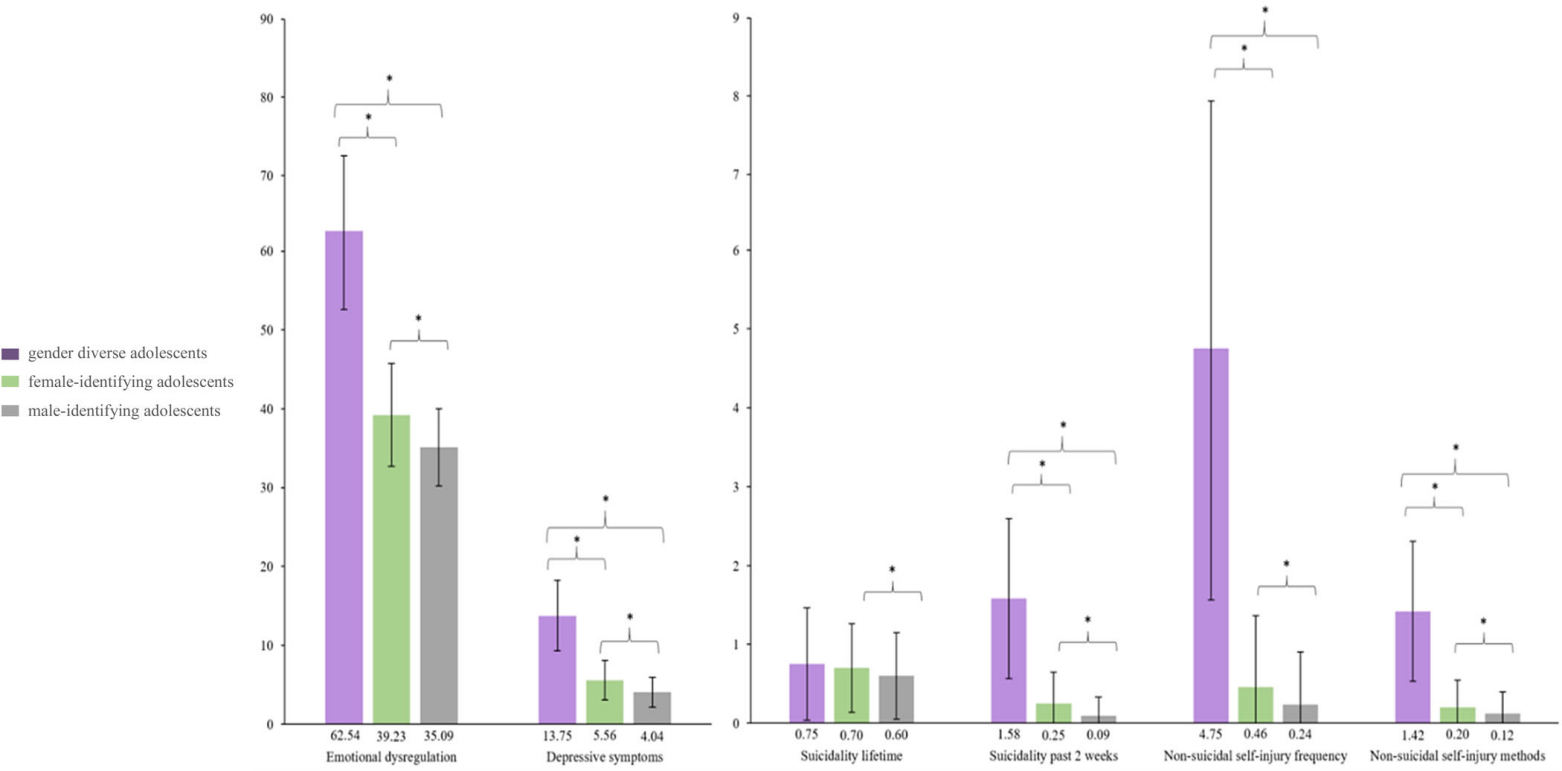
Means of emotional dysregulation, depressive symptoms, suicidality lifetime, suicidality past 2 weeks, non‐suicidal self‐injury frequency, and non‐suicidal self‐injury methods for gender‐diverse, cisgender female and cisgender male adolescents. *indicates significant group comparisons with *p* < 0.05. Error bars represent standard deviation of the mean (SD). The colors of the bar plots reflect the group affiliation of the participants: gender‐diverse adolescents (purple), female‐identifying adolescents (green), male‐identifying adolescents (gray).

Only with regard to lifetime suicidality did no significant differences emerge between gender‐diverse and female‐identifying and male‐identifying adolescents. All comparisons are displayed in Figure 1. For details, see also Table S1 in the supporting information.

## 4 Discussion

Our analyzes revealed significantly higher levels of EmD, depressive symptoms, NSSI lifetime prevalence and frequency, and significantly more NSSI methods in gender‐diverse adolescents compared to their female‐identifying and male‐identifying peers. Although lifetime suicidality did not differ between the groups, gender‐diverse adolescents reported significantly higher levels of suicidality in the past 2 weeks compared to female‐identifying and male‐identifying adolescents.

Our data are consistent with previous research suggesting that gender‐diverse adolescents are a high‐risk group for mental health problems compared to their cisgender peers [9]. Despite this vulnerability, gender‐diverse youth remain underrepresented in prevention research and have received limited attention in early detection and intervention efforts [10]. While some promising approaches exist—such as digital interventions that provide safe spaces for identity exploration [11] and programs emphasizing intersectional youth rights approaches [12]—evidence‐based prevention measures specifically tailored to gender‐diverse adolescents remain scarce.

### 4.1 Implications for Prevention

The elevated symptom burden observed in gender‐diverse adolescents necessitates both universal and targeted prevention approaches. Universal strategies should incorporate comprehensive mental health literacy programs and school‐based anti‐bullying interventions, given the higher victimization rates among sexual and gender minority youth [13]. Targeted interventions require gender‐specific modules addressing minority stress processes, including coping with discrimination and internalized stigma [8].

However, implementation faces substantial barriers. Structural challenges include healthcare access limitations, provider discrimination, and inadequate training for school personnel [14]. Additionally, while isolated progress has been made in validating some screening instruments across gender identities [15], screening measures validated for gender‐diverse adolescent populations are still scarce. These barriers are compounded by data security concerns, particularly regarding involuntary disclosure risks in electronic health records when collecting sensitive gender identity information [16].

### 4.2 Strengths and Limitations

The limitations include the small number of gender‐diverse adolescents and the cross‐sectional design, which precludes causal conclusions. Further, the questionnaires were predominantly developed and normed using female‐identifying and male‐identifying samples, which is why only isolated data points are available for gender‐diverse adolescents (DSHI [17]; PSS [18]; DERS [19]. Although the PHQ‐9 has been administered to gender‐diverse adolescents, no validation studies or normative values exist for this population either [20], limiting comparability with other studies. Moreover, gender identity was assessed using a single questionnaire item with three response options (male, female, diverse), which presents several important limitations. This approach does not provide insight into specific gender identities such as transgender or nonbinary. More critically, some binary transgender adolescents may have selected “male” or “female” in alignment with their gender identity and were consequently misclassified as cisgender, potentially leading to underrepresentation of gender‐diverse participants and measurement bias. To account for this limitation, we use the terms “male‐identifying” and “female‐identifying” adolescents throughout this study rather than assuming cisgender identity. Additionally, this limited assessment approach restricts our understanding of minority stressors related to gender diversity (e.g. stigmatization, rejection [21]. The generalizability of our findings is inherently limited to school‐enrolled adolescents and does not extend to out‐of‐school youth or clinical populations, who may differ systematically in mental health outcomes. Nevertheless, the questionnaire approach also represents an advantage, as adolescents may be more likely to disclose their gender identity via questionnaire than during an interview. Taken together, however, we do not believe that these limitations question the essence of our findings, that is, strongly increased symptom severity in several clearly defined areas of psychopathology, including emotional dysregulation, suicidality, depressiveness, and NSSI.

### 4.3 Future Perspectives

Moving forward, three critical research priorities emerge. First, developing and validating gender‐sensitive assessment instruments specifically normed for gender‐diverse adolescents is essential for accurate identification of mental health needs and treatment outcomes. Second, longitudinal studies examining protective and risk factors unique to gender‐diverse youth trajectories will inform more effective prevention and intervention timing and targets. Finally, adapting established prevention measures through participatory approaches that actively involve gender‐diverse youth in program development will ensure interventions are culturally relevant and acceptable to this population. Such inclusive research will provide the foundation for prevention programs that consider the full spectrum of gender experiences, ultimately ensuring all young people have equitable opportunities for improved mental health outcomes.

## 5 Conclusion

The inclusion of adolescents identifying as gender diverse in research will lay the foundation for determining suitable approaches for prevention measures and developing future prevention programs that consider the variety of gender non‐conforming and gender‐diverse youth, thus giving all young people the same opportunities for improved mental health.

## Author Contributions

**Sabrina Mittermeier:** conceptualization, writing – original draft, writing – review and editing, data curation, formal analysis, visualization. **Sarah Franke:** conceptualization. **Linda M. Bonnekoh:** validation, writing – review and editing. **Georg Romer:** validation. **Marcel Romanos:** funding acquisition. **Nikolaus Kleindienst:** conceptualization, methodology, validation. **Arne Buerger:** conceptualization, supervision, funding acquisition, project administration, writing – review and editing.

## Conflicts of Interest

The authors declare no conflicts of interest.

## Transparency Statement

The lead author Sabrina Mittermeier affirms that this manuscript is an honest, accurate, and transparent account of the study being reported; that no important aspects of the study have been omitted; and that any discrepancies from the study as planned (and, if relevant, registered) have been explained.

## Supporting information


**Supporting Tables 1:** Sample characteristics and group comparisons of gender‐diverse and female‐/male identifying adolescents.

## Data Availability

As this is health‐related data of minors and this data is particularly worthy of protection under data protection law, we cannot make it accessible.
