# Supplementary material for: Urgent Need to Focus on Gender‐Diverse Adolescents in Mental Health Prevention Research: A Cross‐Sectional Comparative Study
Source: Health Sci Rep. 2025 Nov 12;8(11):e71500. doi: 10.1002/hsr2.71500 (PMC12611714; doi:10.1002/hsr2.71500)
Supplement: Supplementary file 1 — Supporting Tables 1: Sample characteristics and group comparisons of gender‐diverse and female‐/male identifying adolescents. [file HSR2-8-e71500-s001.docx]

Supplements: Table 1: Sample characteristics and group comparisons of gender-diverse and female-/male identifying adolescents.

|  | Gender-diverse  adolescents | | | Female-identifying adolescents | | | Male-identifying adolescents | | | Comparison between gender-diverse and female-identifying adolescents | | | Comparison between gender-diverse and male-identifying adolescents | | | Comparison between female- and male-identifying adolescents | | |
| --- | --- | --- | --- | --- | --- | --- | --- | --- | --- | --- | --- | --- | --- | --- | --- | --- | --- | --- |
|  | n | *Mdn* | *IQR* | n | *Mdn* | *IQR* | n | *Mdn* | *IQR* | *U* | *p adj* | *r* | *U* | *p adj* | *r* | *U* | *p adj* | *r* |
| Age in years | 13 | 12.75 | 11.75-12.83 | 1102 | 12.25 | 11.75-12.83 | 1045 | 12.41 | 11.92-12.92 | 5986.5 | .924 | 0.03 | 6182.5 | >.99 | 0.02 | 522344.0 | < .001*** | 0.08 |
| EmD | 13 | 64 | 42-80 | 1102 | 37 | 29-48 | 1044 | 33 | 28-40 | 2488.0 | <.001*** | 0.12 | 1556.5 | <.001*** | 0.15 | 675111.0 | < .001*** | 0.15 |
| Depressive symptoms | 12 | 16.5 | 5.25-19.25 | 1099 | 4 | 2-8 | 1038 | 3 | 1-6 | 3064.0 | <.001*** | 0.10 | 2267.5 | <.001*** | 0.12 | 667941.5 | < .001*** | 0.15 |
| Suicidality | | | | | | | | | | | | | | | | | | |
|  | n | *M* | *SD* | n | *M* | *SD* | n | *M* | *SD* |  |  |  |  |  |  |  |  |  |
| *Lifetime* | 12 | 1.58 | 2.02 | 1097 | 0.25 | 0.8 | 1038 | 0.09 | 0.49 | 6978.5 | n.s. | 0.01 | 6332.5 | n.s. | <0.01 | 598518.5 | .044* | 0.05 |
| *Past 2 weeks* | 12 | 0.75 | 1.42 | 1097 | 0.7 | 1.13 | 1038 | 0.6 | 1.09 | 3931.0 | <.001*** | 0.13 | 3333.0 | <.001*** | 0.23 | 611984.5 | <.001*** | 0.14 |
| NSSI | | | | | | | | | | | | | | | | | | |
| *frequency* | 12 | 4.75 | 6.37 | 1099 | 0.46 | 1.81 | 1040 | 0.24 | 1.33 | 3748.0 | <.001*** | 0.15 | 3366.5 | <.001*** | 0.2 | 591698.0 | .007** | 0.07 |
| *methods* | 12 | 1.42 | 1.78 | 1099 | 0.2 | 0.69 | 1040 | 0.12 | 0.55 | 3823.0 | <.001*** | 0.14 | 3424.0 | <.001*** | 0.19 | 591374.5 | .009** | 0.06 |
|  | n | abs (%) |  | n | abs (%) |  | n | abs (%) |  | Fisher’s exact test | *p* | ϕ​​ | Fisher’s exact test | *p* | ϕ​​ | χ² | *p* | *Cramer’s V* |
| *Lifetime prevalence* | 12 | 6 (50) |  | 1099 | (132) 12.01 |  | 1040 | (99)  9.52 |  |  | <.001*** | 0.14 |  | <.001*** | 0.18 | 7.046 | .008** | 0.06 |

*Mdn* = median, *IQR* = Interquartile range, *M* = mean, *SD* = standard deviation, *U* = Mann-Whitney U test statistics, χ² = Chi-square test statistics, *p adj* = Bonferroni-adjusted *p*-value, EmD = Emotional dysregulation, NSSI = Non-suicidal self-injury, NSSI frequency = frequency of NSSI in the last year, NSSI methods = number of NSSI methods used in the last year, abs = absolute number, * indicates *p* < .05, ** indicates *p* < .01, *** indicates *p* < .001, n.s. indicates non-significance. Due to highly skewed distributions with many zero values, suicidality and NSSI measures are reported as mean ± standard deviation, while other variables are reported as median (IQR).
